# Metabolic heterogeneity, networks, and biomarkers of drug-induced liver injury

**DOI:** 10.1016/j.jpha.2025.101496

**Published:** 2025-11-13

**Authors:** Xian Ding, Hongchuan Liu, Qingrong Qiu, Kongcai Zhu, Xiaohong Zhu, Rui Zhao, Ting Hu, Yuan Sun, Zhuoling An

**Affiliations:** a Beijing Chao-Yang Hospital, Capital Medical University, Beijing, 100020, China; b Beijing Youan Hospital, Capital Medical University, Beijing, 100069, China

**Keywords:** Drug-induced liver injury, Metabolomics, Acylcarnitine, Mendelian randomization, Biomarker

## Abstract

Drug-induced liver injury (DILI) represents a major adverse drug reaction with significant clinical implications. The diversity of causative drug agents, incomplete understanding of pathogenic mechanisms, and absence of specific diagnostic biomarkers pose substantial challenges for DILI diagnosis and clinical management. This study aimed to characterize the metabolic heterogeneity across different types of DILI and identify high-specificity metabolic biomarkers for DILI classification. A multicenter targeted metabolomics study was conducted on 516 serum samples collected from 200 patients with DILI and 221 healthy controls. We characterized the metabolic dynamics throughout DILI progression, with significant disruptions presented in glutathione, fatty acid, and carnitine metabolism. By characterizing and comparing the metabolic profiles among antibiotics-, herbs-, non-steroidal anti-inflammatory drugs-, and statins-DILI patients, we constructed four drug-specific metabolic networks of DILI based on the metabolic coordination between metabolites. Notably, the elevated long-chain acylcarnitines (such as C18:1 Car and C16:2 Car) distinctively underlie herb-DILI's pathological progression. In monocrotaline-induced liver injury mouse models, hepatic carnitine acyltransferase II (Cpt2) mRNA expression was suppressed. Further, two-sample Mendelian randomization supported a causal relationship between C18:1 Car and total bilirubin levels. Finally, we developed a 10-metabolite classifier to distinguish between different DILI subtypes using machine learning algorithms, yielding accuracies of 0.915 and 0.904 on two independent test sets. These findings enhance the understanding of the metabolic heterogeneity in DILI and provide evidence supporting the use of responsive metabolic traits for the clinical diagnosis and treatment of DILI.

## Highlights

• Metabolites' change trajectory and dynamics throughout DILI's disease progress were illustrated.
• Metabolic networks of antibiotics-, herbs-, NSAIDS-, and statins-DILI were constructed based on metabolic coordination.
• Elevated long-chain acylcarnitines potentially contribute to progression of herbs-DILI via Cpt2.
• Mendelian randomization revealed the casual effect between C18:1 Car and TBIL.
• A 10-metabolite model for DILI identification was developed with accuracies of 0.915 and 0.904.

## Graphical abstract

**Figure:**
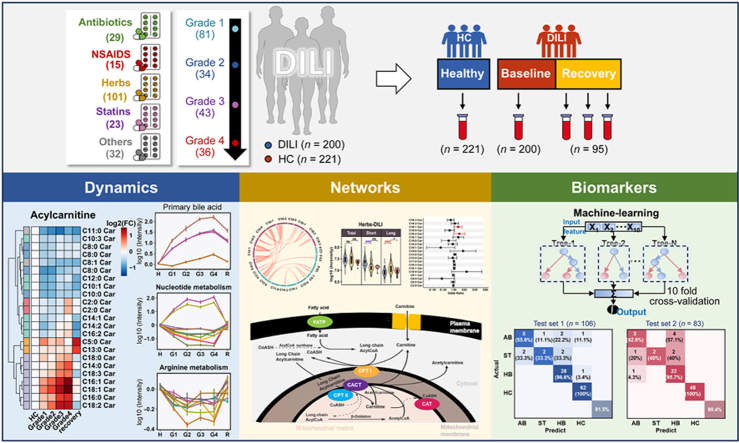

## 1 Introduction

Drug-induced liver injury (DILI) is a clinically significant adverse drug reaction, and its global incidence has been steadily increasing [1]. DILI represents a major contributor to both acute and chronic liver diseases worldwide, accounting for approximately 50% of all acute liver failure cases [2]. To date, thousands of drugs have been implicated in hepatotoxicity, including antibiotics [3], non-steroidal anti-inflammatory drugs (NSAIDS) [4], anti-tuberculosis drugs, anti-tumor drugs [5], herbal products [6], and many drug excipients. Getting more in-depth into the mechanistic heterogeneity underlying DILI progression is critical for developing targeted therapies tailored to different DILI subtypes.

The clinical manifestations of DILI can range from asymptomatic, mild, and non-specific biochemical changes to acute liver failure. Currently, the gold standard for DILI diagnosis is liver biopsy with a premier of exclusion from other causes of liver damage, which is often highly challenging, and patients are often misdiagnosed. Although conventional liver function tests and the Roussel Uclaf Causality Assessment Method (RUCAM) score have been widely applied as diagnostic tools for DILI [7,8], their limited specificity and sensitivity hinder the accurate prognosis prediction and clinical decision-making in the subsequent clinical course of DILI. Furthermore, due to the vast diversity of hepatotoxic agents, the pathogenesis of DILI has not yet been clarified, which leads to failure in clinical management. Therefore, there is an urgent need to identify non-invasive specific biomarkers or characterization methods for DILI.

The advancement of omics technology prompts the investigation of more effective biomarkers for DILI diagnosis. Several novel and promising biomarker candidates have been identified [9], such as glutamate dehydrogenase, keratin 18, sorbitol dehydrogenase, glutathione S-transferase, cytochrome P450, and miR-122. However, most of these biomarkers are protein- or RNA-based, with limited representation at the metabolite level. Metabolomics, being highly sensitive to pathological changes and external stimuli, has emerged as a powerful tool for biomarkers discovery in disease diagnosis and prognostic assessment [10]. Indeed, metabolites and lipids, such as bile acids and poly-unsaturated fatty acids [11], have been shown to correlate with DILI progression.

Nevertheless, many metabolite-disease associations remain observational, lacking causal evidence. Mendelian randomization (MR), a genetic epidemiological approach, addresses this limitation by leveraging genetic variants as instrumental variables to investigate causal relationships between exposures and outcomes [12]. A landmark 2023 metabolome-wide genome-wide association study (GWAS) mapped genetic variants associated with 1400 human blood metabolites [13], providing crucial insights into the genetic foundation of blood metabolomics. A recent MR study revealed the mediation effects linking gut microbiota, metabolites, and DILI, offering potential biomarkers for risk stratification and mechanisms elucidation [14].

To address these gaps, this study was designed to investigate the metabolic landscape and identify biomarkers of DILI by integrating targeted metabolomics [15], transcriptomic analysis, and MR analysis. Firstly, we characterized the metabolic trajectories during both the disease progression and recovery phases of DILI. Next, we compared the metabolic profiles of DILI patients induced by four distinct drug classes (antibiotics, herbs, NSAIDS, and statins), subsequently constructing drug-specific metabolic networks. We further dissected acylcarnitine metabolism differences among these subtypes, with validation of carnitine acyltransferase (Cpt) expression levels performed using an external monocrotaline-induced liver injury mouse transcriptome dataset (GSE102150) [16]. A two-sample MR analysis was performed to explore the causal effects between the levels of 18 blood acylcarnitine species, DILI, and total bilirubin (TBIL). Ultimately, leveraging machine learning algorithms, we developed a high-performance metabolomic classifier using ten metabolites, which could identify different DILI subtypes.

## 2 Materials and methods

### 2.1 Ethical statement

The Ethics Committee of Beijing Youan Hospital, Capital Medical University approved this study (LL-2019-155-K), and the requirement for informed written consent was waived because the samples were retrospectively collected. The Ethics Committee of Beijing Chao-Yang Hospital, Capital Medical University approved this study (2015-Research-39 and 2017-Research-150), and each patient provided informed consent before sample collection. This study followed the Declaration of Helsinki, and all procedures were performed in compliance with relevant laws and institutional guidelines.

### 2.2 Chemicals and reagents

Mass spectrometry (MS) grade acetonitrile and high-performance liquid chromatography (HPLC) grade isopropyl alcohol were purchased from Thermo Fisher Scientific (Pittsburgh, PA, USA). Formic acid (FA) was purchased from TEDIA Co., Inc. (Fairfield, OH, USA). Ultrapure water was prepared with a Milli-Q Direct 8 water purification system (Millipore, Bedford, MA, USA). Stable isotope labelled internal standards (IS), including thymine-d4, valine-d8, phenylalanine-d8, 17-hydroxyprogesterone-d8, docosahexaenoic acid-d5, cholic acid-d4, chenodeoxycholic acid-d4 and glycocholic acid-d4, were purchased from Cambridge Isotope Laboratories (Cambridge, MA, USA).

### 2.3 Inclusion/exclusion criteria for participant enrollment

A total of 200 patients diagnosed with DILI were recruited in this study. 146 patients with DILI were retrospectively recruited from Beijing YouAn Hospital between April 2015 and April 2019, and 54 patients with DILI were recruited from Beijing Chao-Yang Hospital between December 2017 and April 2019, with the inclusion/exclusion criteria as follows. Inclusion criteria for DILI: (1) Age >18 years old; (2) Have a definite medication history and have different degrees of digestive system symptoms such as loss of appetite, fatigue, upper abdominal discomfort, nausea and vomiting within 1–4 weeks after medication; (3) The laboratory tests indicate liver function damage, such as the increase of serum alanine aminotransferase (ALT), aspartate transaminase (AST), alkaline phosphatase (ALP), and total bilirubin (TBIL), which is clinically diagnosed as DILI. Exclusion criteria for DILI: (1) Suffering from viral hepatitis B or C and other types of viral hepatitis, autoimmune liver disease, alcoholic liver disease, non-alcoholic fatty liver disease, hemochromatosis, Wilson disease, antitrypsin deficiency; (2) Suffering from compensatory or decompensated cirrhosis; (3) Combined with diabetes, gout, phenylketonuria; (4) Previously or currently suffering from malignant tumors; (5) Women who are pregnant or breastfeeding; (6) There is no clear record of drugs that cause DILI; (7) Patients after organ or bone marrow transplantation; (8) Complicated with severe diseases of other systems.

A total of 221 healthy individuals were included from the physical examination center, Beijing Chao-Yang Hospital, from April 2018 to April 2019. Inclusion criteria for healthy controls: (1) Liver function parameters (such as ALT, AST, ALP, and TBIL) were within the upper limit of normal (ULN); (2) Age >18 years old. Exclusion criteria for the healthy controls: (1) Suffering from any organic diseases or neuropsychiatric disorders; (2) Having suffered from malignant tumors; (3) Women who are pregnant or breastfeeding.

### 2.4 Clinical definition

According to the drug types that cause DILI, all the 200 patients with DILI were divided into antibiotics-DILI (AB, *n* = 29), NSAIDS-DILI (NS, *n* = 15), herbs-DILI (HB, *n* = 101), statins-DILI (ST, *n* = 23), and others DILI group (*n* = 32). Among 101 patients in the HB group, 62 cases (61.4%) took traditional Chinese medicine decoctions with unclearly traced ingredients. 13 cases (12.9%) took traditional Chinese medicine decoctions or Chinese patent medicines containing *Polygonum multiflorum* Thunb. One patient took traditional Chinese medicine decoctions with Psoraleae Fructus, and one patient took traditional Chinese medicine decoctions with *Angelica dahurica*. The other 25 patients used several different Chinese patent medicines, such as Xinyuan Capsules and Pingtang Huanyisu. Among those HB patients with clearly identified causes, *Polygonum multiflorum* Thunb is the primary type of herb in DILI.

According to the Chinese Guidelines for the Management of DILI, 194 DILI patients were graded according to severity, and six patients could not be graded because of missing information. Grade 1 (G1, *n* = 81): serum ALT and/or ALP are recoverably elevated, TBIL <2.5 × ULN (2.5 mg/dL or 42.75 μmol/L), and international normalized ratio (INR) < 1.5; Grade 2 (G2, *n* = 34): serum ALT and/or ALP are elevated, TBIL ≥2.5 × ULN (2.5 mg/dL or 42.75 μmol/L), or INR ≥1.5 without elevated TBIL; Grade 3 (G3, *n* = 43): serum ALT and/or ALP are elevated, TBIL ≥5 × ULN (5 mg/dL or 85.5 μmol/L), with or without INR ≥1.5; Grade 4 (G4, *n* = 36): serum ALT and/or ALP are elevated, TBIL ≥10 × ULN (10 mg/dL or 171 μmol/L) or daily rise ≥1.0 mg/dL (17.1 μmol/L), INR ≥2.0 or prothrombin activity (PTA) < 40%.

According to the Chinese Guidelines for the Management of DILI, 141 DILI patients were categorized into hepatocellular (*n* = 92), cholestatic (*n* = 15), and mixed type (*n* = 34) by the R ratio (R = (ALT/ULN)/(ALP/ULN)). 1) hepatocellular DILI: R ≥ 5; 2) cholestatic DILI: R ≤ 2; 3) mixed DILI: 2 < R < 5. 59 patients could not be clinically categorized because of missing information.

The healthy control (HC, *n* = 221) group was clinically defined as volunteers with liver function parameters (including ALT, AST, ALP, and TBIL) that were within the ULN: 1) serum ALT <40 U/L for female, and serum ALT <50 U/L for male; and 2) serum AST <35 U/L for female, and serum AST <40 U/L for male; and 3) serum ALP <100 U/L for female, and serum ALP <125 U/L for male; and 4) serum TBIL <21 μmol/L for female, and serum TBIL <26 μmol/L for male.

### 2.5 Serum sample collection

A total of 516 serum samples were collected from 421 individuals for targeted metabolomics analysis, which were composed of 200 DILI patients and 221 healthy individuals. Among these samples, 200 serum samples were collected before they received treatments (baseline stage) from the DILI patients, and 95 additional serum samples were gathered during the patient's hospitalization (recovery stage), during which they received treatments with liver protection medicine. 221 serum samples were collected from healthy individuals. The serum sample collection procedure was as follows: the blood was drawn in the tubes after standard diagnostic tests. Then, it stored at 4 °C for several hours to allow the blood to coagulate. Next, it was centrifuged at 3000 rpm for 10 min at 4 °C to prepare serum. The serum was frozen at −80 °C until metabolite extraction.

### 2.6 Metabolite extraction

50 μL of serum, 140 μL of methanol solution, and 10 μL of mixed IS solution containing thymine-d4, valine-d8, phenylalanine-d8, 17-hydroxyprogesterone-d8, docosahexaenoic acid-d5, cholic acid-d4, chenodeoxycholic acid-d4, and glycocholic acid-d4 at 400 ng/mL were mixed for protein precipitation. The mixture was then vortexed for 2 min with a Vortex-Genie 2 vortex mixer (Scientific Industries, Bohemia, NY, USA), followed by centrifugation at 13,800 rpm and 4 °C for 10 min with a Heraeus Fresco 21 refrigerated microcentrifuge (Thermo Scientific, Waltham, MA, USA). The supernatants were collected for high-performance liquid chromatography-tandem mass spectrometry (HPLC-MS/MS) analysis. The pooled quality control (QC) samples were prepared with equal mixing of all test serum samples, and the QC samples were pre-prepared using the same procedures as the test serum samples.

### 2.7 Calibration solution preparation

The standard mixture solution was prepared using all the standards listed in the previous publication [15]. The working solution was gradually diluted with methanol to prepare standard curves with concentrations of 0.2, 0.5, 2, 5, 20, 50, 100, 200, 500, 1,000, 2,000, and 5000 ng/mL. Next, 50 μL of water, 50 μL of the standard mixture, 90 μL of methanol, and 10 μL of mixed IS solution containing thymine-d4, valine-d8, phenylalanine-d8, 17-hydroxyprogesterone-d8, docosahexaenoic acid-d5, cholic acid-d4, chenodeoxycholic acid-d4, and glycocholic acid-d4 at 400 ng/mL were mixed. Then, the preparation procedures for standard curve samples were the same as those for serum samples, which were mentioned in Section 2.6.

### 2.8 LC-MS/MS-based targeted quantitative metabolomics analysis

Targeted quantitative metabolomics analysis was conducted according to the previously reported method [15]. The metabolomics experiment was conducted using a Spark Holland liquid chromatography system (Spark Holland, Emmen, The Netherlands) in tandem with an API 5500 mass spectrometer coupled with Turbo Ion Spray™ source (AB Sciex, Concord, Canada). Metabolite separation was performed utilizing an Xselect HSS T3 column (150 mm × 2.1 mm, 3.5 μm; Waters, Milford, MA, USA) with mobile phases of water containing 0.1% formic acid (A) and acetonitrile/isopropyl alcohol in a 7:2 (*v/v*) ratio (B). Gradient elution settings were as follows (flow rate = 0.5 mL/min): 0–4 min, 1%–10% B; 4–8 min, 10%–50% B; 8–15 min, 50%–80% B; 15–25 min, 80 %–100% B; 25–27 min, 100 % B; 27–27.1 min, 100%–1% B; 27.1–29 min, 1% B. The column oven temperature was maintained at 20 °C, and the injection volume was 5 μL.

Mass spectrometry settings were as follows: source temperature, 600 °C; ion source gas 1, 60 psi; ion source gas 2, 60 psi; curtain gas, 40 psi; electrospray voltage, −4500 V for negative scan and 5500 V for positive scan. All the metabolites were analyzed in a single injection using negative and positive modes with scheduled multiple reactions monitoring.

Targeted metabolomics analysis was performed on the 516 serum samples. The pooled serum QC samples were evenly distributed throughout the sample analysis process to monitor the system stability, with an injection frequency of 1/20 (one QC sample was analyzed after 20 analytical samples).

### 2.9 Targeted metabolomics data processing

Raw MS data files were processed using Multi-Quant software (version 3.0.2). Firstly, the peak area of metabolites and ISs were extracted. Next, the linearity calibration curves between the peak area ratio of metabolite and IS and the actual concentrations were fitted using a weighted least-squares method with a weighting factor of 1/x^2^. Then, the absolute concentrations of metabolites were calculated using the fitted linearity calibration curve. For some acylcarnitines and fatty acids without standards, their relative concentrations were calculated using the peak area ratio of an analyte to the corresponding IS. Finally, the absolute concentrations and relative concentrations of 143 metabolites were obtained and output into files (.xlsx) for subsequent statistics analysis.

### 2.10 C-means clustering analysis

Firstly, 141 metabolites with *P* < 0.05 by two-sided Kruskal–Wallis test between HC, G1, G2, G3, G4, and recovery (R) group were selected. Next, the expressions of those selected metabolites among each group were used for fuzzy c-means clustering, executed through the Mfuzz package (version 2.58.0). Then, those metabolites were clustered into different clusters based on expression trends across groups of HC, G1, G2, G3, G4, and R. Finally, the alteration trajectories of representative metabolites across groups of HC, G1, G2, G3, G4, and R were depicted with heatmaps and line plots (mean ± standard error of the mean (SEM)).

### 2.11 Correlational network analysis

Firstly, a two-sided Kruskal–Wallis test followed by a Dunnett multiple comparisons test was performed between HC versus AB group, HC versus HB group, HC versus NS group, and HC versus ST group, respectively. Next, the expressions of those metabolites with *P* < 0.05 and fold change >1.5 or <0.667 were selected for Pearson correlation analysis. Finally, the significant correlations between metabolites (*P* < 0.001 & |r| > 0.5) were input into Cytoscape software (version 3.10.1) to construct the drug (AB, HB, NS, and ST) specific correlation networks for visualization. Besides, the top 5 most connected metabolites (with the top 5 most degrees) were listed in every network.

### 2.12 Transcriptome data analysis

A transcriptome dataset from the liver tissues of monocrotaline-induced liver injury mouse models [16] was downloaded from the open-source public database of Gene Expression Omnibus (https://www.ncbi.nlm.nih.gov/geo/query/acc.cgi?acc=GSE102150). The differentially expressed genes were screened with *P* adjusted with false discovery rate (FDR) < 0.05 and fold change > 1.2 or < 0.833, executed by DESeq2 package. Pathway enrichment analysis (adjusted *P* < 0.005) was executed with the differentially expressed genes using the clusterProfiler package.

### 2.13 MR analysis integrating GWAS and metabolome data

Two sample MR analysis was performed to explore the causal effects of 18 blood carnitine levels and DILI or TBIL, which was exceuted by ‘TwoSampleMR’ package. All the GWAS datasets were obtained from the IEU OpenGWAS, NHGRI-EBI GWAS catlog. and FinnGen database. GWAS data for DILI included 186 cases and 213,592 controls, which were diagnosed based on the International Classification of Diseases (ICD10: K71). GWAS datasets for TBIL (ebi-a-GCST90013872, ebi-a-GCST90014012) included 388,303 individuals, and GWAS data for 18 blood carnitines included individuals ranging from 6287 to 8273. Five methods (inversevariance-weighted (IVW), MR-Egger, weighted median, simple mode, and weighted mode) were used to evaluate and validate causal effects. Cochran's *Q* statistic was calculated to evaluate heterogeneity. The MR-Egger intercept was performed to evaluate pleiotropy. Only a causal effect with *P*-value of <0.05 and without heterogeneity and pleiotropy (corresponding *P*-value of >0.05) was identified as a significant effect.

### 2.14 Statistical analysis

All data were subjected to analyses using R software (version 4.3.2). Specifically, a two-sided Kruskal–Wallis test was used when comparing three or more groups. A two-sided Kruskal–Wallis test followed by Dunnett multiple comparisons was used when comparing two groups among multiple groups, and a two-sided Wilcoxon test was used when comparing two groups for unpaired samples. Missing values were imputed with a multiple imputation method using the Mice package. Pathway analysis was conducted in an open-source website of MetaboAnalyst 4.0 (https://www.metaboanalyst.ca/), and the differential abundance score was applied to represent the tendency for a pathway to have increased levels of metabolites relative to a control group. The significant protein-enriched pathways were identified as the pathways with *P* < 0.05. Pearson correlation coefficients between metabolites and metabolites or between metabolites and clinical variables were calculated using the Hmisc package. Heatmaps were constructed using the Heatmap package. Bubble plots, box plots, and bar plots were implemented using the ggplot2 package. Circos plot was constructed using the Circliz package. Sanky diagram was constructed using the ‘ggalluvial’ package. Statistical significance was set as ^∗^*P* < 0.05, ^∗∗^*P* < 0.01, ^∗∗∗^*P* < 0.001, and ^∗∗∗∗^*P* < 0.0001.

### 2.15 Machine-learning

A metabolomic prediction classifier for DILI diagnosis was built using a machine-learning algorithm with the Caret package. The participants were randomly stratified sampling into a discovery dataset (*n* = 206) and two test sets (test set 1, *n* = 120; test set 2, *n* = 95). First, we developed a series of machine-learning models including gradient boosting machine (GBM), support vector machine, k-means, eXtreme Gradient Boosting, and artificial neural network on the discovery dataset, and the GBM model was selected as the best-performance model according to the 10-fold cross-validated accuracy and Kappa coefficient. Next, we selected the ten optimal metabolites in terms of variable importance to the GBM model. Then, a GBM model was trained with the ten selected metabolites in the discovery dataset. The GBM model was constructed by constantly splitting feature nodes and building new trees, with a goal of reducing prediction residual and promoting classification accuracy. The parameters were optimized using a grid search approach based on 10-fold cross-validation according to accuracy and kappa, and the final model parameters were as follows: shrinkage = 0.2, interaction depth = 4, n minobsinnode = 1, n trees = 45. Afterward, the diagnostic model was applied to test set 1 and test set 2 to evaluate the model performance. The model was evaluated with a receiver operating characteristic curve (ROC) and confusion matrix. The final predicted value for DILI diagnosis was output in the forms of the HC and DILI prediction probabilities for each sample, which was computed by the weighted summary of prediction results of all trees. Individuals would be identified as the group with a higher predicted value (>0.5).

To further identify the DILI types that are caused by different drugs, including antibiotics, statins, and herbs, another 10-metabolite prediction classifier was built based on the GBM algorithm. First, ten optimal metabolites were selected using GBM in terms of the variable importance on the discovery dataset (AB, *n* = 13; ST, *n* = 12; HB, *n* = 49; HC, *n* = 111). Then, a GBM model was trained with the ten selected metabolites in the discovery dataset, and the parameters were optimized using a grid search approach based on 10-fold cross-validation. The final model was trained under the following parameters: shrinkage = 0.2, interaction depth = 8, n minobsinnode = 0.5, n trees = 8. Afterward, the diagnostic model was applied to test set 1 (AB, *n* = 9; ST, *n* = 6; HB, *n* = 29; HC, *n* = 62) and test set 2 (AB, *n* = 7; ST, *n* = 5; HB, *n* = 23; HC, *n* = 48) to evaluate the model performance. The model was evaluated using the area under receiver operating characteristics curve (AUC), accuracy, sensitivity, specificity, and F1 scores. Finally, the probability of HC, AB, ST, and HB was computed as the sum predicted probabilities of all the trees; individuals would be identified as the group with the highest predicted values.

## 3 Results

### 3.1 Study design and sample characteristics

**Fig. 1:**
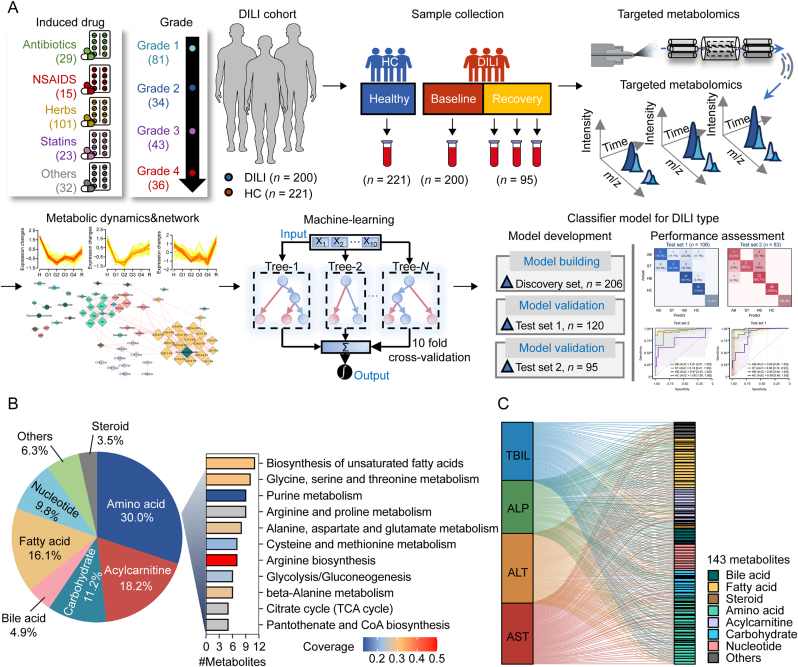
Patient enrollment, study design, and metabolome overview of drug-induced liver injury (DILI) study. (A) Schematic overview of patient enrollment and the study design. (B) The structure classes and top 11 enriched pathways by the 143 metabolites detected by targeted metabolomics. (C) The Pearson correlations (*P* < 0.05) between clinical indicators (such as total bilirubin (TBIL), alkaline phosphatase (ALP), alanine aminotransferase (ALT), and aspartate transaminase (AST)) and 143 metabolites. HC: healthy control; AB: antibiotics-DILI; HB: herbs-DILI; ST: statins-DILI.

The overall workflow of this study is illustrated in Fig. 1A. Specifically, 200 DILI patients and 221 healthy individuals were recruited in this study, with demographic characteristics presented in Table S1. According to the causative drug types, DILI patients were divided into AB (*n* = 29), NS (*n* = 15), HB (*n* = 101), ST (*n* = 23), and others (*n* = 32) groups. According to the severity of liver damage, 194 DILI patients were graded into G1 (*n* = 81), G2 (*n* = 34), G3 (*n* = 43), and G4 (*n* = 36) groups. 141 DILI patients were categorized into hepatocellular (*n* = 92), cholestatic (*n* = 15), and mixed type (*n* = 34). Afterward, 200 serum samples of baseline and 95 serum samples of recovery were collected from DILI patients, along with 221 serum samples from healthy individuals. The metabolic profiles of these serum samples were then acquired using a targeted metabolomics approach based on LC-MS/MS technology. The raw data is presented in Table S2. In total, 143 metabolites were detected, encompassing amino acids, nucleotides, acylcarnitines, carbohydrates, bile acids, fatty acids, and steroids, which covered 17 distinct pathways (*P* < 0.05; Fig. 1B). The relative standard deviation (RSD) values of all metabolites in QC samples were below 20%, indicating high repeatability and reproducibility in the targeted metabolomics data. Remarkably, significant correlations were observed between metabolites and clinical parameters (Fig. 1C). These results indicated that the serum metabolic profile of DILI patients reflected the changes in clinical phenotype. Next, we compared the metabolic landscape between HC and DILI patients at baseline and recovery stages and further explored the metabolic correlational networks across different DILI subtypes. Finally, we developed the DILI diagnostic model consisting of 10 metabolites using the GBM algorithm. The model's performance in distinguishing AB, HB, and ST patients from HC was evaluated on two test sets (test set 1 and test set 2).

### 3.2 Metabolic dynamics throughout the progression and recovery evolution process of DILI

**Fig. 2:**
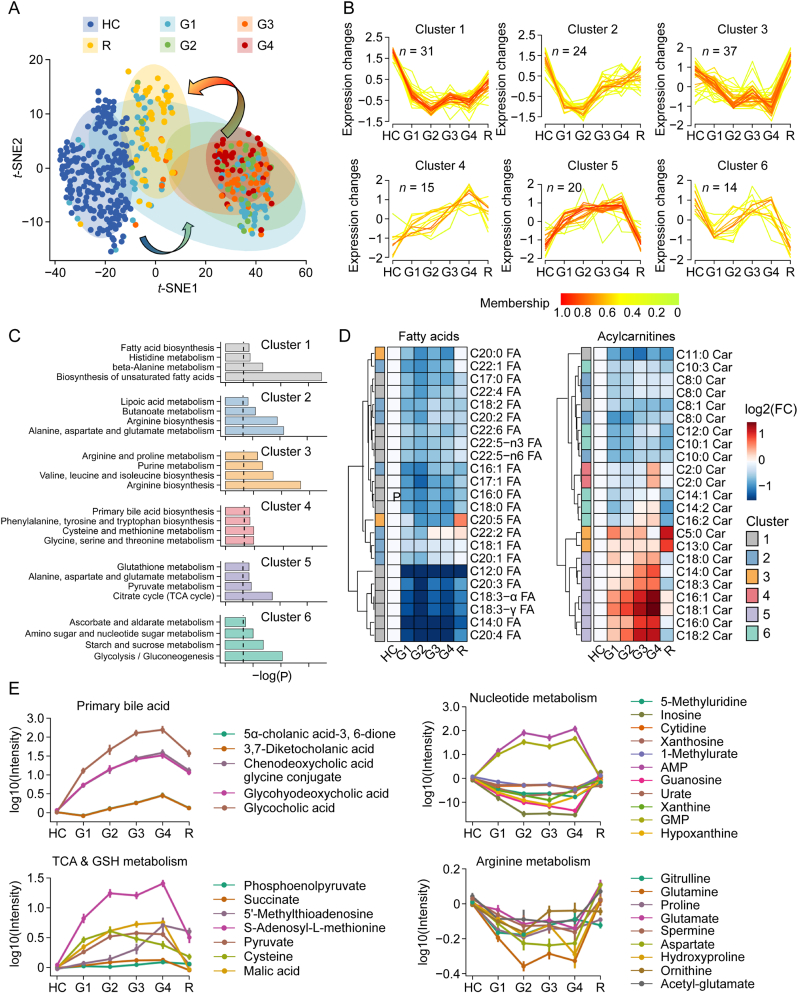
Metabolic dynamics throughout the progression of drug-induced liver injury (DILI). (A) *t*-distributed stochastic neighbour embedding (*t*-SNE) analysis of each serum sample from the targeted metabolomics data (healthy control (HC), *n* = 221; Grade 1, *n* = 81; Grade 2, *n* = 34; Grade 3, *n* = 43; Grade 4, *n* = 36; Recovery, *n* = 95). (B) C-means clustering of metabolic trajectories throughout the aggravation and recovery of DILI using the change trends of differential metabolites. (C) Kyoto Encyclopedia of Genes and Genomes (KEGG) pathway enrichment analysis based on the metabolites of clusters 1, 2, 3, 4, 5, and 6. (D) Heatmap of free fatty acids (left) and acylcarnitines (right) in DILI samples representing the intensities relative to HC. (E) The average levels of the representative metabolites change against the DILI aggravation and recovery in the clusters of primary bile acid biosynthesis, nucleotide metabolism, tricarboxylic acid (TCA) cycle, glutathione (GSH) metabolism, and arginine metabolism. The intensities were normalized to the intensities of HC. The dots represent the mean of normalized intensities, and the bars represent the standard error of mean (SEM). G1: Grade 1; G2: Grade 2; G3: Grade 3; G4: Grade 4; R: Recovery; GMP: guanosine 5-monophosphate; AMP: adenosine 5-monophosphate; FC: fold change.

To characterize the serum metabolic reprogramming during the progression and recovery of DILI, we performed the differential analysis among HC, G1–G4 DILI, and the recovery group. T-distributed stochastic neighbour embedding (*t*-SNE) analysis results (Fig. 2A) revealed distinct clustering between HC and DILI groups. Notably, G3–G4 samples exhibited greater separation from HC than G1–G2 samples. However, recovery-phase samples clustered intermediately, suggesting a gradual metabolic normalization. These findings indicate that the metabolic profiles are susceptible to the progression of DILI, which provided a potential molecular basis for the clinical diagnosis of DILI. Using c-means clustering, we defined six distinct molecular clusters reflecting the dynamic metabolic patterns (Fig. 2B). Interestingly, nearly half of the metabolites exhibited uni-directional behaviours, which were increased or decreased from G1 to G4 until reaching their peaks or troughs immediately before recovering phase, such as metabolites in clusters 1, 4, and 5. Cluster 1 was enriched in the unsaturated fatty acid biosynthesis pathway, cluster 4 was enriched in primary bile acid biosynthesis, glycine, serine and threonine metabolism, and cysteine and methionine metabolism, and cluster 5 was enriched in citrate cycle and pyruvate metabolism (Fig. 2C). In contrast, metabolites in clusters 2, 3, and 6 displayed irregular fluctuations, which were mainly enriched in arginine metabolism, glycolysis and gluconeogenesis metabolism, and purine metabolism (Fig. 2C).

Specifically, long-chain polyunsaturated fatty acids declined across the progression of liver injury, whereas primary bile acids steadily increased (Figs. 2D and E). Acylcarnitines exhibited intricate dynamics: short-chain species decreased, while long-chain species accumulated (Fig. 2D). Given the liver's central role in fatty acid metabolism and the critical function of acylcarnitines in mitochondrial β-oxidation, these metabolic patterns suggest an incomplete inhibition of mitochondrial fatty acids β-oxidation in DILI, potentially contributing to microvesicular steatosis and liver failure [17]. Such disorders of fatty/bile acid homeostasis were one of the underlying mechanisms of DILI progression [11]. Glutamate, cysteine, 5′-methylthioadenosine, and S-adenosyl-L-methionine are precursor metabolites for synthesizing glutathione. Except for glutamate, these metabolites' expression levels were elevated during the progressions of liver injury (Fig. 2E), indicating the disruption of redox metabolism. Mitochondrial oxidative stress has been reported as the target of hepatotoxicity of many drugs [18]. In contrast with the increasing patterns displayed by guanosine 5-monophosphate (GMP) and adenosine 5-monophosphate (AMP), other nucleotides such as inosine, xanthosine, guanosine, and cytidine displayed stepwise decreasing patterns from G1 to G4 and reached levels comparable to HC (Fig. 2E). Inosine was a potential immunomodulatory agent with potent anti-inflammatory effects [19]. Arginine metabolism is considered to be an essential metabolic regulator for macrophage polarization and inflammatory response, which has been reported to be related to acute liver injury through L-arginine-nitric oxide-cyclic GMP signaling [20]. Collectively, these results demonstrated that the metabolism of fatty acids, acylcarnitines, bile acids, nucleotides, glutathione, and arginine constituted the whole metabolic dynamics for regulating the progress of DILI.

### 3.3 Metabolic network of liver injury induced by different drugs

The mechanisms underlying DILI are highly complex. To elucidate the metabolic regulatory differences among different DILI types, we compared the metabolic profiles of hepatocellular, cholestasis, and mixed DILI (Fig. S1A). A total of 78, 67, and 51 discriminant metabolites were identified in the hepatocellular, cholestasis, and mixed DILI patient groups (Figs. S1B and C). These metabolites revealed widespread dysregulation in key pathways, including biosynthesis of unsaturated fatty acid, amino acid metabolism, and purine metabolism (Fig. S1D). Notably, amino acid metabolism emerged as the main metabolic characteristics that distinguish between the hepatocellular, cholestasis, and mixed DILI types, such as phenylalanine, glutamate, cystine, alanine, and aspartate (Fig. S1E).

**Fig. 3:**
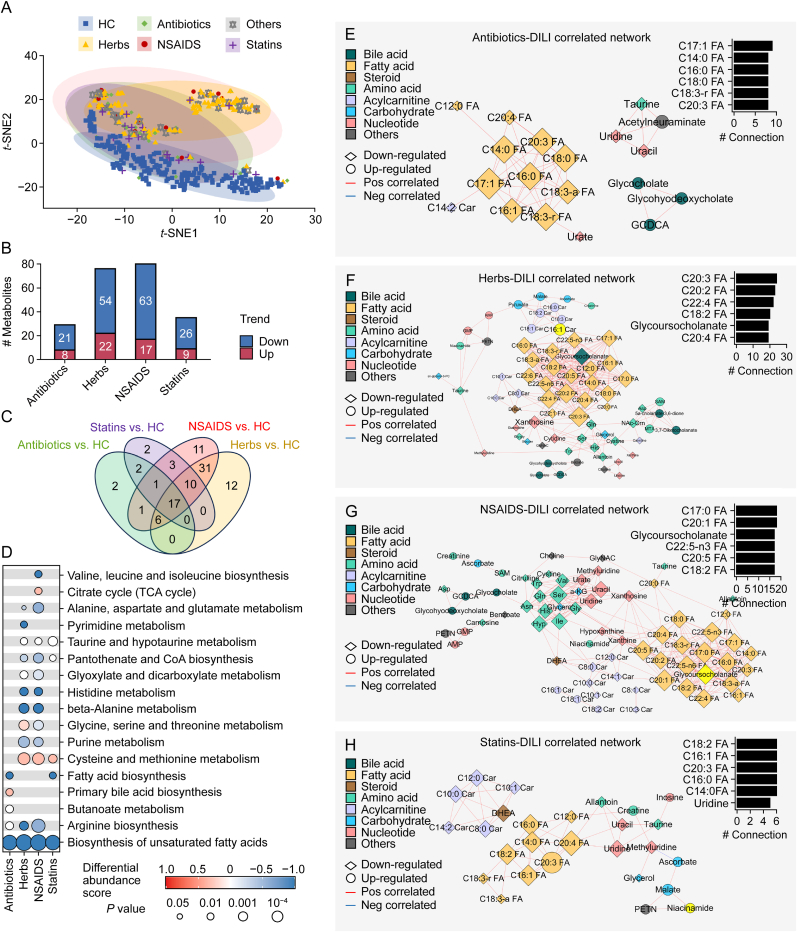
Metabolic network of drug-induced liver injury (DILI) induced by different drugs. (A) *t*-distributed stochastic neighbour embedding (*t*-SNE) analysis of each sample from the serum-targeted metabolomics data (healthy control (HC), *n* = 221; antibiotics-DILI, *n* = 29; herbs-DILI, *n* = 101; non-steroidal anti-inflammatory drugs (NSAIDS)-DILI, *n* = 15; statins-DILI, *n* = 23; others, *n* = 32). (B) Summary of the significant up-regulated and down-regulated metabolites between HC and antibiotics-DILI, herbs-DILI, NSAIDS-DILI, and statins-DILI groups. (C) Venn plot indicating the differentially expressed metabolites between HC and antibiotics-DILI, herbs-DILI, NSAIDS-DILI, and statins-DILI groups. (D) Kyoto Encyclopedia of Genes and Genomes (KEGG) pathways enriched using differentially expressed metabolites in (B–C). (E–H) Correlation networks of differentially expressed metabolites in antibiotics-DILI (E), herbs-DILI (F), NSAIDS-DILI (G), and statins-DILI (H) patients (*P* < 0.001 & |r| > 0.5).

We next analyzed the metabolic differences across AB, HB, NS, and ST groups. t-SNE analysis demonstrated overlapping metabolic profiles between AB and ST samples, whereas HB exhibited distinct clustering (Fig. 3A). NS samples displayed greater dispersion, reflecting higher interindividual variability. Compared with the HC group, 21, 54, 68, and 26 metabolites were up-regulated in the AB, HB, NS, and ST groups, respectively; In contrast, 8, 22, 17, and 9 metabolites were down-regulated (Fig. 3B). Venn plot demonstrated that 45.4% (20/44) metabolites were shared by AB and ST group, and 69.6% (64/92) metabolites were shared by HB and NS group, only 28.0% (23/82) metabolites were shared by AB and HB group (Fig. 3C). These results indicated that more similar metabolic patterns were shared between AB and ST group or between HB and NS group. Pathway analysis highlighted consistent down-regulation in the biosynthesis of unsaturated fatty acids across four groups (Fig. 3D). HB and NS generated more profound metabolic disturbances, with the down-regulations of pyrimidine metabolism, purine metabolism, histidine metabolism, alanine metabolism, and arginine biosynthesis alongside the up-regulations of citrate cycle, cysteine and methionine metabolism. In contrast, AB and ST exhibited alterations in fatty acid biosynthesis, primary bile acid biosynthesis, and cysteine and methionine metabolism.

To identify the functional metabolite groups associated with DILI, we performed drug-specific correlation analysis on the profiles of those differentially expressed metabolites. We first categorized those differentially expressed metabolites into eight classes using existing structural and biological information. Interestingly, metabolites within the same classes and with consistent expression trends clustered together in the correlation matrices. Based on the correlation relationship, we constructed drug-specific correlation networks to explore the potential regulatory relationships in DILI (Figs. 3E–H). The network's topology indicated that dense interactions occurred between inter- and intra-metabolite classes, and the decreased fatty acids occupied the center positions in the networks of all DILI types. This aligns with reports implicating that fatty acid β-oxidation reprogramming is involved in the progress and prognosis of liver injury induced by several different drugs, including Tripterygium Wilfordii [21], acetaminophen, amiodarone, ibuprofen, linezolid, and nucleoside reverse transcriptase inhibitors [17]. Notably, acylcarnitines exhibited unique correlation patterns in HB group. Although decreased short-chain acylcarnitines correlated with fatty acids, steroid hormones, and nucleotides in all four groups, HB group specifically showed that increased long-chain acylcarnitines were strongly linked to carbohydrates and fatty acids. Accumulation of acylcarnitines reflects impaired mitochondrial β-oxidation in the liver [21], which can lead to microvesicular steatosis, hypoglycemia, and liver failure [17]. Collectively, these findings demonstrate that despite dynamic metabolite alterations during DILI progression, a highly coordinated metabolic regulatory network underlies the pathology of DILI. The distinct acylcarnitine coordination in further highlights drug etiology-specific metabolic rewiring.

### 3.4 Elevated long-chain acylcarnitines contribute to the progress of herbs induced DILI

**Fig. 4:**
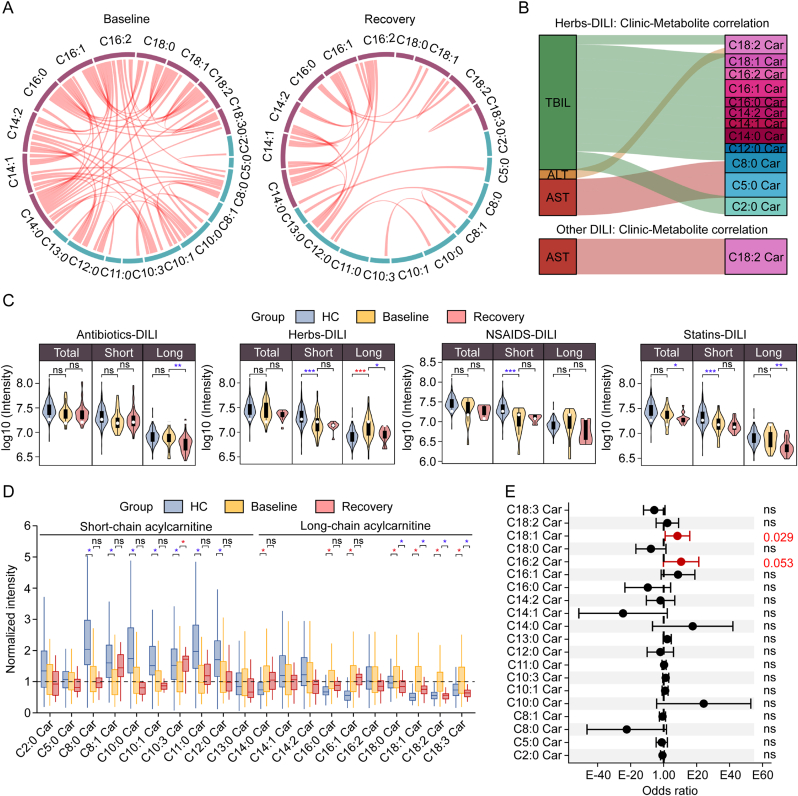
Elevated long-chain acylcarnitines are beneficial to the progress of herbs-drug-induced liver injury (DILI). (A) The significant inter-correlations between metabolites of short-chain and long-chain acylcarnitines (*P* < 0.001 & |r| > 0.5) in herbs-DILI patients at the baseline and recovery time. (B) The significant correlations (*P* < 0.05) between the expressions of acylcarnitines and clinical indicators (such as total bilirubin (TBIL), alanine aminotransferase (ALT), and aspartate transaminase (AST)) in herbs-DILI (top) and all other DILI (bottom) patients. (C) Levels of acylcarnitines in serum samples between healthy control (HC) and DILI (herb-, antibiotics-, non-steroidal anti-inflammatory drugs (NSAIDS)-, and statins-DILI) patients at baseline and recovery time. ^∗^*P* < 0.05; ^∗∗^*P* < 0.01; ^∗∗∗^*P* < 0.001; ns, not significant. (D) Levels of individual acylcarnitine in serum samples between HC and herbs-DILI patients at baseline and recovery time. The data was normalized to the levels of baseline. ^∗^*P* < 0.05; ns, not significant. (E) Odd ratios of acylcarnitines related to the herbs-DILI status (two-sided z-test of logistic regression). ns, not significant. Error bars represent 95% confidence intervals.

To obtain insights into the metabolic regulatory role of acylcarnitines in HB patients, we examined the metabolic coordination between acylcarnitines at baseline and recovery time. Pearson correlation analysis identified 60 positively correlated acylcarnitines at baseline, 51 of which were long-chain acylcarnitines. In contrast, only 19 positive correlations were observed during recovery phase (Fig. 4A). Correlation analysis between metabolites and clinical indicators demonstrated that long-chain acylcarnitines were highly correlated with TBIL in HB patients compared to other DILI subtypes (Fig. 4B). It is speculated that HB patients are more likely to be characterized by elevated TBIL levels, as shown in Fig. S2.

We next quantified the changes of total, short-chain, and long-chain acylcarnitines in the baseline and recovery time across HB, AB, NS, and ST patients (Fig. 4C). Total acylcarnitine levels showed no significant differences between HC and DILI patients (except in ST patients); short-chain acylcarnitines were significantly reduced in nearly all DILI patients at baseline, but with no notable differences during recovery time. Conversely, long-chain acylcarnitines were markedly elevated in HB patients at baseline but sharply declined during the recovery time, displaying a pattern distinct from short-chain acylcarnitines and other DILI subtypes. Further comparative analysis of individual acylcarnitines showed that HB patients had higher baseline levels of long-chain species, including C14:0 Car, C16:0 Car, C16:1 Car, C18:0 Car, C18:1 Car, C18:2 Car, and C18:3 Car (Fig. 4D). Among these, C18:0 Car, C18:1 Car, C18:2 Car, and C18:3 Car decreased significantly during recovery time. In contrast, for short-chain species, only C10:3 Car showed significant alterations at both time points.

To assess the contribution of acylcarnitines to herbs induced DILI progression, we constructed a logistic regression model and calculated the odds ratio for each acylcarnitine (Fig. 4E). C18:1 Car and C16:2 Car had odds ratios greater than 1, suggesting that elevated levels of these long-chain acylcarnitines may promote herbs induced DILI pathogenesis. Given that acylcarnitine is an indispensable metabolite hub for transferring long-chain acyl-CoA from cytosol to mitochondrial matrix for β-oxidation, which is regulated by carnitine acyltransferase I and II (Cpt1 and Cpt2) (Fig. S3A); the observed down-regulation of fatty acids and up-regulation of long-chain acylcarnitines in HB patients suggest dysregulations of Cpt1 and Cpt2.

To further investigate the role of carnitines in herbs induced DILI progression, we analyzed a liver transcriptome dataset (GSE102150) from the monocrotaline (MCT)-induced liver injury mouse models [16]. MCT is a pyrrolizidine alkaloid derived from the medicinal herb Crotalaria ferruginea Grah and is often used as an anti-tumor agent. Comparative mRNA expression analysis between MCT-treated (dose: 330 mg/kg) and control mice groups revealed enrichment of differentially expressed genes in fatty acid degradation pathway (Fig. S3B). Among the Cpt1 isoforms (CPT1a, CPT1b, CPT1c) and Cpt2, Cpt1a and Cpt2 were down-regulated in the liver tissues of the MCT group, whereas Cpt1c was up-regulated (Fig. S3C). Collectively, these results demonstrate that acylcarnitines serve as significant metabolic markers reflecting liver injury status. Notably, long-chain acylcarnitines were substantially elevated at baseline but decreased during recovery in HB patients, suggesting their pathogenic relevance. Our data further indicate that elevated long-chain (rather than short-chain) acylcarnitines may contribute to herbs induced DILI progression, highlighting long-chain carnitine metabolism as a potential therapeutic target for herbs associated DILI.

### 3.5 MR analysis reveals the causal effects of C18:1 Car on TBIL

**Fig. 5:**
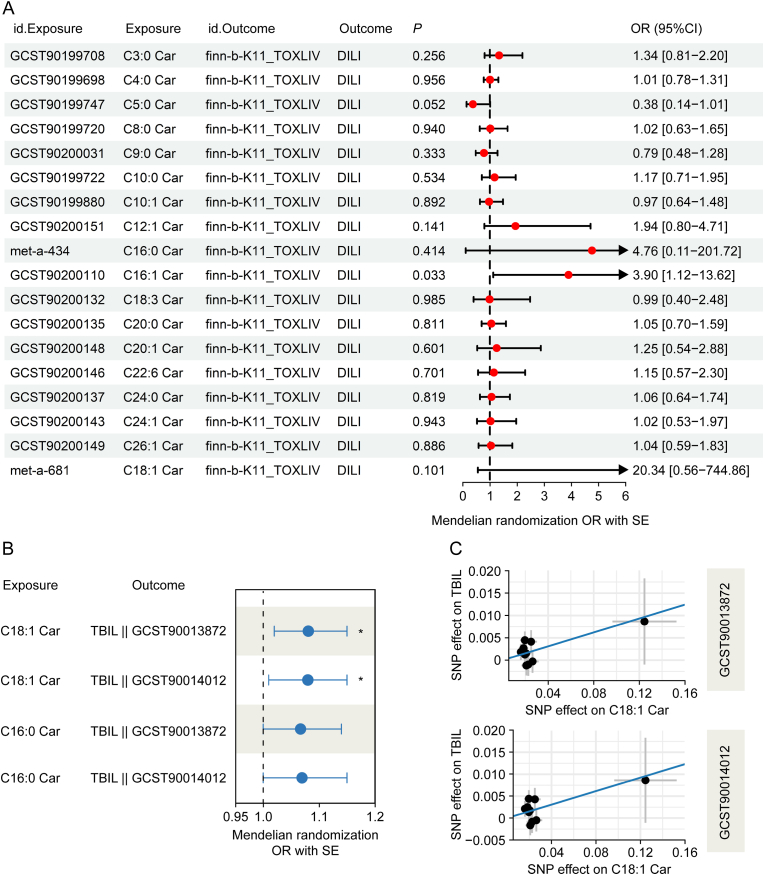
Mendelian randomization (MR) analysis results between acylcarnitines, drug-induced liver injury (DILI), and total bilirubin (TBIL). (A) Forest plot indicating MR analysis results between the 18 acylcarnitine species and DILI. Error bars represent odds ratio (OR) ± standard error (SE). (B) Forest plot indicating MR analysis results between the C18:1 Car, C16:0 Car and TBIL. Error bars represent OR ± SE. (C) Scatter plots of the MR models with a potential causal relationship between C18:1 Car and TBIL of GCST90013872 (top) and GCST90014012 (bottom) genome-wide association study (GWAS) data. 95%CI: 95% confidence interval.

To further investigate the causal relationship between acylcarnitines and DILI, we performed a two-sample MR analysis using GWAS data for 18 blood acylcarnitines as exposure and DILI GWAS data as outcome. The results indicated a causal relationship between long-chain carnitine (C16:1 Car) and DILI (*P* < 0.05, Fig. 5A). Given that HB patients exhibited higher TBIL levels than other DILI subtypes (Fig. S2), we further conducted MR analysis between the 18 acylcarnitines and TBIL. The results revealed a positive causal relationship between long-chain acylcarnitine (C18:1 Car) and TBIL (Fig. 5B). Specifically, an increase of 1 standard deviation in C18:1 Car resulted in an 8% increase in TBIL (OR = 1.08, 95% CI: 0.18 to 0.80), as shown in Fig. 5C. The data further suggests that elevated C18:1 Car levels exert a significant causal effect on TBIL elevation. Targeting the metabolism of C18:1 Car could facilitate the recovery of TBIL-elevated DILI.

### 3.6 Machine learning-based metabolomic classifier identifies drug sources of liver injury

**Fig. 6:**
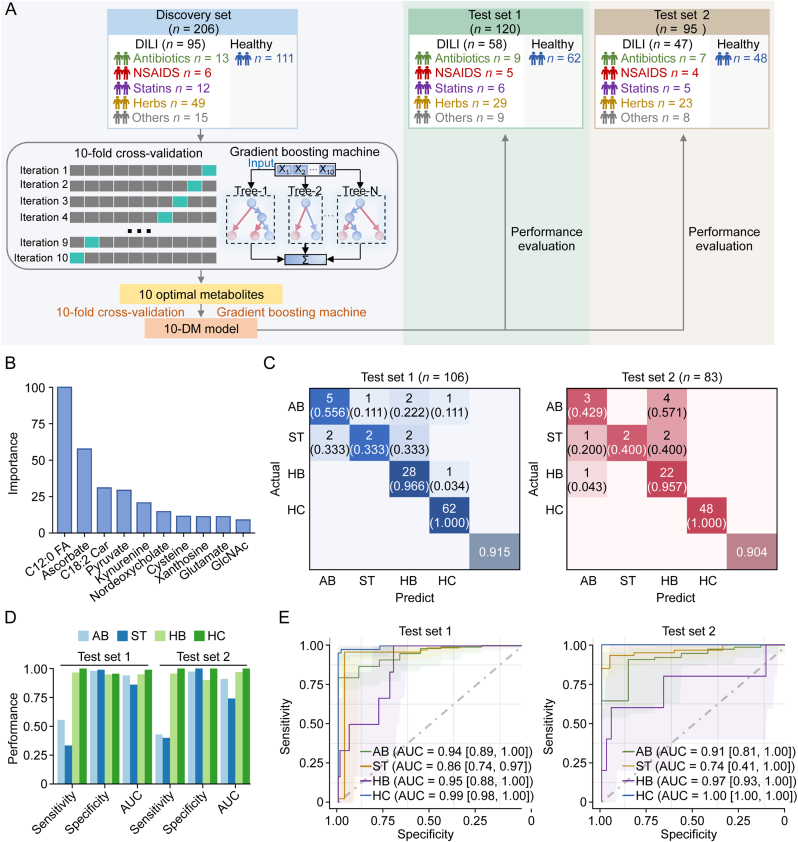
Machine learning-derived prediction model based on serum metabolome for identifying drug-induced liver injury (DILI) from different drug sources. (A) Design of the modeling workflow. Gradient boosting machine algorithms were adopted for feature selection and model training on discovery set. The 10-differential metabolites (DM) model was validated on test sets 1 and 2. (B) Contribution of the ten metabolites to the 10-DM model. The ten metabolites are C12:0 FA, ascorbate, C18:2 Car, pyruvate, kynurenine, nordeoxycholate, cysteine, xanthosine, glutamate, and N-acetyl-D-glucosamine (GlcNAc). (C) Confusion matrix of the 10-DM model for discriminating healthy controls (HC), antibiotics-DILI (AB), herbs-DILI (HB), and statins-DILI (ST) patients on test sets 1 and 2. (D) The sensitivity, specificity, and area under the curve (AUC) of the 10-DM model on test sets 1 and 2. (E) The receiver operating characteristic (ROC) curve and AUC value for identifying HC, AB, HB, and ST DILI patients on test sets 1 and 2. A 95% confidence interval was computed with 2000 stratified bootstrap replicates.

Leveraging the acquired reprogrammed metabolic profiles, we developed DILI diagnostic models. Using the GBM algorithm, we selected 10 optimal metabolites in terms of variable importance and trained a classifier model for the discrimination of DILI and HC (Figs. 6A and S4A). The model demonstrated robust performance on the validation test sets 1 and 2 (Fig. S4B), achieving an AUC of 1 (accuracy: 0.983, sensitivity: 0.966, specificity: 1) in the test set 1 and AUC of 1 (accuracy: 0.989, sensitivity: 0.979, specificity: 1) in the test set 2 (Figs. S4C and D).

Next, we developed an another metabolomic classifier using a similar algorithm pipeline (Fig. 6A) to identify the specific drug sources of liver injury (antibiotics, statins, and herbs). The screened biomarker panel included 10 metabolites: C12:0 FA, ascorbate, C18:2 Car, pyruvate, kynurenine, nordeoxycholate, cysteine, xanthosine, glutamate, and N-Acetyl-D-glucosamine (GlcNAc). The relative abundance plots confirmed significant differences in these ten metabolites between HC and DILI (AB, ST, and HB) subgroups (Fig. S5). Each metabolite contributed relatively evenly to this 10-metabolite diagnostic model (10-DM model), with C12:0 FA, ascorbate, and C18:2 Car being the three most significant contributing metabolites (Fig. 6B). Previous studies on Tripterygium Wilfordii-induced liver injury have consistently reported the accumulated serum level of C18:2 Car [21] and reduced serum levels of free fatty acids [22]. Given that mitochondrial fatty acid oxidation is a key metabolic pathway for hepatic energy production, its impairment—often induced by drugs—can lead to hepatic steatosis, marked by dysregulation of lipid metabolism. Ascorbate is widely recognized for its hepatoprotective effects against drug-induced hepatotoxicity. The decreased ascorbate levels had been observed in the liver tissues of targeted therapy-induced liver injury [23], and the increased levels have been reported in the urine of rats with Aurantio-Obtusin-induced liver injury [24].

To further evaluate the model's diagnostic efficacy across different DILI etiologies, we validated the model on test sets 1 and 2. On the test set 1 and 2, the model achieved high accuracies of 0.915 and 0.904 (Fig. 6C), with AUCs of 0.99/1.00 (sensitivity: 1.000/1.000, specificity: 0.955/1.000) for HC, AUCs of 0.95/0.97 (sensitivity: 0.966/0.957, specificity: 0.948/0.900) for HB, AUCs of 0.86/0.74 (sensitivity: 0.333/0.400, specificity: 0.990/1.000) for ST, and AUCs of 0.94/0.91 (sensitivity: 0.556/0.429, specificity: 0.979/0.974) for AB patients (Fig. 6D and E). The lower sensitivity in distinguishing AB and ST groups may stem from their similar and overlapped metabolic profiles. However, when these two groups were merged, this model could achieve a higher sensitivity of 0.66 and 0.6 for a combined AB + ST group on test sets 1 and 2. Collectively, our machine-learning-based metabolomic classifier demonstrates superior discriminatory power in identifying DILI patients from diverse drug exposures, offering promising potential for clinical application in DILI diagnosis.

## 4 Discussion

This study used a targeted metabolomics approach to characterize the serum metabolic profiles in patients with DILI. We identified dynamic metabolic alterations corresponding to the disease progression. Although there are many studies on the metabolomics of DILI, few studies focus on the metabolic shifts throughout the aggravating and recovering phases [11]. The gradings on the DILI samples according to the disease severities allowed us to determine a broad set of high-resolution metabolic profiles during DILI progressions. We preliminarily analyzed the metabolic features of DILI with four different grades and further compared the metabolic alterations in the recovery phase after therapeutic intervention: almost all metabolites were observed to be altered during the progression process of DILI (Fig. 2B). Notably, changes in bile acids and fatty acids aligned with prior reports [11,25], validating our findings.

We also identified novel metabolites associated with DILI progression, such as 5-methyluridine, malic acid, and spermine, which have not been adequately studied. Among the dynamically changing metabolites, bile acids, energy metabolites, and REDOX metabolites predominated the increasing metabolites clusters. Elevated bile acids are well-documented to exert effects in DILI and chronic liver disease [25]. Succinate, a mitochondrial signaling molecule, is considered to promote hepatic fibrogenesis by promoting activation, proliferation, and migration and inhibiting apoptosis of hepatic stellate cells [26]. Pyruvate and 5′-methylthioadenosine were reported to attenuate ischemia-reperfusion injury after liver transplantation in rats [27,28]. Fatty acids and arginine metabolites were declined. The arginine/citrulline cycle was observed to be closed in the non-alcoholic fatty liver disease rat model, characterized by decreased arginase production and low availability of NOS arginine substrate by hepatocytes [29]. Nucleotides and acylcarnitines exhibited divergent trends: C18:2 Car, AMP, and GMP increased with DILI severity, whereas C8:0 Car, C11:0 Car, inosine, and cytidine were decreased. Strikingly, most metabolites normalized after therapeutic intervention, underscoring their sensitivity to DILI and potential as biomarkers.

The mechanism of DILI is complex, as the drugs that induce liver injury are diverse. Previous studies seldom focused on comparing metabolic patterns of liver injury induced by different drugs. To this end, we analyzed and compared metabolic profiles of AB, HB, ST, and NS groups. Biosynthesis of unsaturated fatty acids was universally perturbed, while some distinct metabolic characteristics were drug-specific. For example, purine metabolism and taurine metabolism were changed in HB and NS patients, and pyrimidine metabolism was changed in HB patients. Bile acid metabolism was up-regulated in AB patients (Fig. 3D). These findings align with prior reports. For example, total bile acid was reported to be increased in rifampicin-induced injury in HepG2 cells, and tauroursodeoxycholic acid (TUDCA) could alleviate liver injury via increasing bile acid transporters expression and enhancing the Nrf2-mediated adaptive response [30]. Purine metabolism and pyrimidine metabolism (such as xanthine, xanthiosine, and uridine), which were related to DNA damage response, were found to be decreased in the Gardeniae Fructus extract-treated HepG2 cells and rats [31]. Taurine treatment was effective in counteracting APAP-induced hepatic damage, oxidative stress and cellular necrosis [32]. These results indicate the heterogeneity of metabolic characteristics between different drug-induced liver injuries. In addition, we constructed drug-specific metabolic regulatory networks for four types of DILI. Network analysis conduces to get a deeper insight into the interactions between metabolites in different DILI types. Fatty acids, glycoursocholate, and uridine were highlighted as the core metabolites in the drug-specific correlation networks (Figs. 3E–H). The liver is a primary organ for synthesizing and metabolizing fatty acids in the human body. Abundant evidence shows that lipid metabolism is sensitive to any injury in the liver [33,34]. Previous studies reported the link between uridine homeostasis, pyrimidine metabolism [35], and liver lipid metabolism, and uridine homeostasis disruption could induce hepatic microvesicular steatosis and injury. Interestingly, long-chain and short-chain acylcarnitines were observed to show differences in the metabolites’ inter-correlations in HB patients, suggesting a potential distinct carnitine metabolic regulatory mechanism of herbs induced DILI progression.

A notable finding of this study is that the elevated long-chain (rather than short-chain) acylcarnitine metabolism is linked specifically to herbs induced DILI progressions. We observed significantly increased baseline serum levels of long-chain carnitines (such as C18:0 Car, C18:1 Car, and C18:2 Car) in HB patients, which subsequently decreased during the recovery period (Figs. 4C and D). This is an intriguing dynamic metabolic phenomenon that only occurs in HB patients and is different from other types of DILI. Besides, elevated serum levels of C16:2 Car and C18:1 Car appeared particularly associated with herbs induced DILI development (Fig. 4E). MR analysis established further causal effects between long-chain acylcarnitine and DILI, and specifically between C18:1 Car and TBIL. It suggested a distinct metabolic pattern of acylcarnitine existed in herbs induced DILI, which plays a role in regulating TBIL.

Considerable studies have revealed the importance of acylcarnitine in DILI related to herb drugs, such as Triptolide [21], *Polygonum multiflorum* Thunb [36]. and Psoraleae Fructus [37]. The consistency between our study and other independent studies reinforced the presented results. More importantly, we acquired a liver transcriptome dataset from the monocrotaline-induced liver injury mouse and validated the inhibition of Cpt2, explaining the accumulations of long-chain acylcarnitines. Acylcarnitine is an indispensable metabolite hub for mitochondrial β-oxidation of long-chain fatty acids. Previous studies have shown that reduced mitochondrial β-oxidation energy supply may be a potential mechanism leading to liver failure [38]. The severity of liver lesions depends on the residual mitochondrial beta-oxidation flux [17]. Many drugs could induce inhibition of mitochondrial fatty acid oxidation and steatosis [39,40]. For example, acylcarnitines were found to accumulate in blood soon after APAP-induced mitochondrial damage [40]. Hepatic long-chain acylcarnitines significantly increased after oral administration of amiodarone and valproic acid, and the hepatic mRNA levels of Cpt1 and Cpt2 were significantly suppressed [41,42]. Inhibition of Cpt1 was reported to induce mitochondrial injury of hepatocytes and the accumulation of cellular carnitines [43]. Therefore, our findings provide a potential therapeutic intervention by targeting carnitine metabolism for HB patients characterized with elevated TBIL, which warrants further experimental and clinical studies.

Machine learning allowed us to develop a metabolomic classifier using ten metabolites, which could identify the drug sources of DILI. The drugs that cause DILI are complex and diverse, and the lack of reliable specific biomarkers to identify the drug sources of DILI poses challenges for the diagnosis and treatment of DILI. We identified a set of biomarkers (Fig. 6), and these metabolites showed significance between HC, HB, AB, and ST patients. Numerous metabolomics studies have reported blood biomarkers for the evaluation of DILI, and we compared our identified biomarkers with those previous results. Serum C18:2 Car was found to be up-regulated in the serum samples of *Tripterygium wilfordii*-induced liver injury rat models [21]; serum GlcNAc and xanthiosine were dropped in the tripterygium glycoside-induced liver injury rat models [15]. Serum nordeoxycholate was reported to be elevated in the tripterygium glycoside-induced liver injury rat models [15,44] and decreased in the moxifloxacin-induced liver injury rat models [45]. Blood kynurenine was reported to be down-regulated in the Polygoni Multiflori Radix Preparata [46] and tripterygium glycoside [15] induced liver injury models. Elevated pyruvate is commonly seen in the liver diseases related to traditional Chinese medicine toxicology [47]. These reported results were consistent with our results (Fig. S5), which have verified our findings. Furthermore, several previously reported tissue and urinary biomarkers for drug-induced liver injury assessment also proved the accuracy of our findings. For example, C12:0 FA was decreased in both liver tissue and blood samples of triptolide-induced liver injury models [22], and cysteine was elevated in the urine of atorvastatin-induced liver injury rats [48] (Fig. S5). The established machine-learning model demonstrated enhanced diagnostic performance, with improved sensitivity, specificity, and accuracy in determining DILI causation. Several previous studies have been reported to date exploring the biomarkers of DILI. However, most of these studies have focused on DNA, RNA, and proteins as potential biomarkers for DILI [9,49]. Metabolome, as the terminal of molecular phenome, is highly sensitive to DILI status and demonstrates potential for novel biomarkers identification [50]. Therefore, in terms of metabolism and DILI, our work underlies the values of metabolites in predicting DILI.

This study has some strengths. First, the incorporation of serum samples from two independent medical centers enhances the generalizability of our findings. Second, using a targeted metabolomics approach, all the serum metabolites were quantitated with absolute or relative concentrations instead of the peak's relative intensity. Therefore, it decreased the false discoveries and improved the precision and accuracy of our analytical results, which also offered promise for the clinical application of the metabolomic classifier in the future. Third, the study takes advantage of the resources of large public databases, including GWAS and transcriptome public datasets. Multi-dimensional data provides cross-validation of the accuracy of our findings. However, we acknowledge several important limitations. First, the detection coverage of targeted metabolomics remains limited, potentially omitting biologically relevant metabolites. Second, our sample size is still insufficient for initial biomarker discovery. Larger and multi-center patient cohorts will be essential to fully establish the clinical validity of these biomarkers. Future studies with expanded recruitment and untargeted metabolomic profiling are needed to verify the diagnostic potential of our findings across diverse populations and uncover additional metabolic signatures of DILI.

## 5 Conclusions

In summary, this study investigated the metabolic landscape and biomarkers of DILI by integrating targeted metabolomic, transcriptomic analysis and MR analysis. Firstly, we performed targeted metabolomics on 516 serum samples of 200 DILI patients and 221 healthy individuals. The DILI patients were classed by induced-drug resources and liver injury grades. Afterward, we investigated the metabolic change trajectory during the DILI progressions, compared the differences in the metabolic profiles of DILI patients caused by antibiotics, herbs, NSAIDS, and statins, and constructed drug-specific metabolic networks of DILI patients. Particularly, we found the differences in the acylcarnitine metabolism mechanisms of DILI caused by these four types of drugs. Key findings were validated through analysis of an external liver transcriptome dataset from the monocrotaline-induced liver injury mouse models, which confirmed the suppression of Cpt2. Two-sample MR analysis further established the causal effects between C18:1 Car and TBIL. Finally, we developed and validated a machine learning-based metabolomic classifier incorporating ten metabolites, which could accurately discriminate DILI etiologies. This work provides novel insights into the metabolic heterogeneity of DILI and establishes a foundation for metabolomics-guided clinical diagnosis and management of DILI. The identified metabolic signatures and classifiers represent promising tools for improving DILI stratification and personalized treatment approaches.

## CRediT authorship contribution statement

**Xian Ding:** Writing – review & editing, Writing – original draft, Visualization, Investigation, Data curation, Conceptualization. **Hongchuan Liu:** Investigation, Formal analysis, Conceptualization. **Qingrong Qiu:** Resources. **Kongcai Zhu:** Resources. **Xiaohong Zhu:** Resources. **Rui Zhao:** Methodology. **Ting Hu:** Methodology. **Yuan Sun:** Resources. **Zhuoling An:** Writing – review & editing, Supervision, Project administration.

## Declaration of competing interests

The authors declare that they have no known competing financial interests or personal relationships that could have appeared to influence the work reported in this paper.
