# Supplementary material for: Metabolic heterogeneity, networks, and biomarkers of drug-induced liver injury
Source: J Pharm Anal. 2025 Nov 13;16(3):101496. doi: 10.1016/j.jpha.2025.101496 (PMC13014638; doi:10.1016/j.jpha.2025.101496)
Supplement: Multimedia component 1 [file mmc1.docx]

**Supporting Information**

**Metabolic heterogeneity, networks, and biomarkers of drug-induced liver injury**

Xian Ding^1#^, Hongchuan Liu^1#^, Qingrong Qiu^2^, Kongcai Zhu^2^, Xiaohong Zhu^2^, Rui Zhao^1^, Ting Hu^1^, Yuan Sun^1^, Zhuoling An^1,*^

1. Beijing Chao-Yang Hospital, Capital Medical University, Beijing, 100020, China.

2. Beijing Youan Hospital, Capital Medical University, Beijing, 100069, China.

#Both authors contributed equally to this paper.

^*^ Corresponding authors:

**Zhuoling An** –Beijing Chao-Yang Hospital, Capital Medical University, Beijing, 100020, China. Email: anzhuoling@163.com; anzhuoling@bjcyh.com;

**Contents**

[**Table S1.** Demographics and baseline characteristics of DILI patients. S-3](#_Toc198293203)

[**Fig.S1.** The metabolic profiles of hepatocellular, cholestatic, and mixed DILI. S-4](#_Toc198293204)

[**Fig.S2.** The levels of total bilirubin (TBIL) in the serum samples of healthy controls, herbs-DILI, and others-DILI patients. S-5](#_Toc198293205)

[**Fig.S3.** Carnitine metabolism in GSE102150 transcriptome dataset. S-6](#_Toc198293206)

[**Fig.S4.** Machine learning-derived prediction model based on plasma metabolome for DILI diagnosis. S-7](#_Toc198293207)

[**Fig.S5.** The expressions of the ten metabolites selected for model development in the serum samples of healthy controls, antibiotics-DILI, herbs-DILI, and statins-DILI patients. S-8](#_Toc198293208)

**Table S1.** Demographics and baseline characteristics of drug-induced liver injury (DILI) patients.

| Characteristics | Healthy  control  （*n* = 221） | DILI patients | | | | |
| --- | --- | --- | --- | --- | --- | --- |
|  |  | Antibiotics  （*n* = 29） | Herb  （*n* = 101） | NSADIS  （*n* = 15） | Statins  （*n* = 23） | Others  (*n* = 32) |
| Age  (mean ± SD, years) | 32.30±10.30 | 54.55±16.79 | 49.05±14.28 | 50.73±9.63 | 53.61±11.76 | 51.63±12.39 |
| Geder  (Male/Female) | 34/187 | 9/20 | 67/34 | 11/4 | 7/16 | 19/13 |
| BMI  (mean ± SD, Kg/m^2^) | 22.70±2.12 | 25.19±4.00 | 24.34±3.86 | 25.66±3.27 | 25.67±5.39 | 24.76±4.20 |
| Grade  (1/2/3/4/NA) | --- | 22/2/2/1/2 | 25/17/29/29/1 | 6/2/4/2/1 | 12/7/2/0/2 | 16/6/6/4/0 |
| Pathological Type  (hepatocellular/ cholestasis/ mixed/ NA) | --- | 5/3/7/14 | 57/9/13/22 | 9/0/2/4 | 7/0/3/13 | 14/3/9/6 |

BMI: Body Mass Index; NA: Not available;


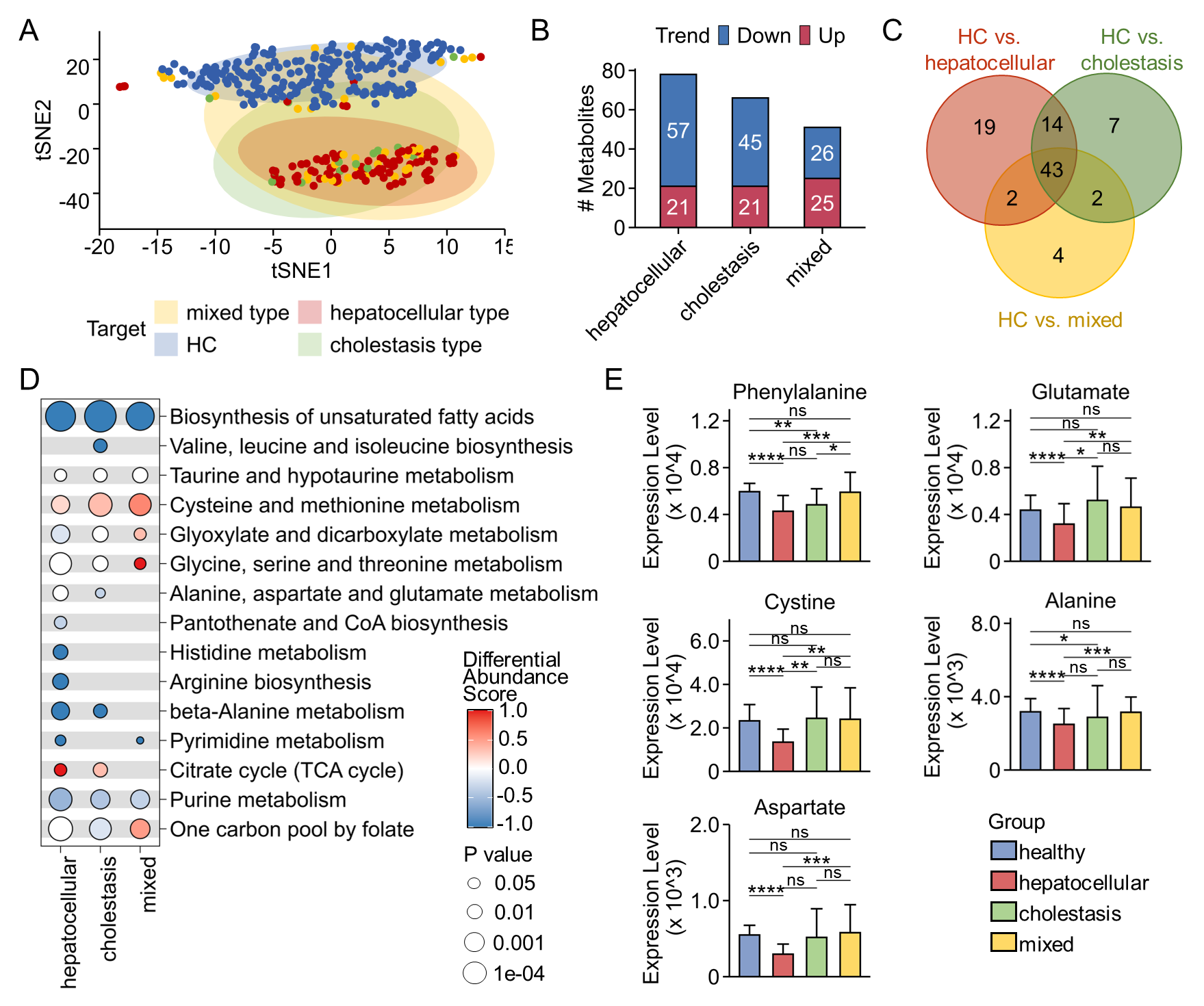


**Fig. S1.** The metabolic profiles of hepatocellular, cholestatic, and mixed drug-induced livery injury (DILI).

(A) T-distributed stochastic neighbour embedding (t-SNE) analysis of each sample from the plasma-targeted metabolomics data (HC (healthy control), *n* = 221; hepatocellular DILI, *n* = 92; cholestatic DILI, *n* = 15; mixed DILI, *n* = 34). (B) Summary of the significant up-regulated and down-regulated metabolites between HC, hepatocellular DILI, cholestatic DILI, and mixed DILI groups. (C) Venn plot indicating the differentially expressed metabolites between HC, hepatocellular DILI, cholestatic DILI, and mixed DILI groups. (D) Kyoto Encyclopedia of Genes and Genomes (KEGG) pathways enriched using differentially expressed metabolites in (B-C). (E) The expressions of phenylalanine, glutamate, cystine, alanine, and aspartate in HC, hepatocellular DILI, cholestatic DILI, and mixed DILI groups. *, *P* < 0.05; **, *P* < 0.01; ***, *P* < 0.001; ****, *P* < 0.0001; ns, not significant.


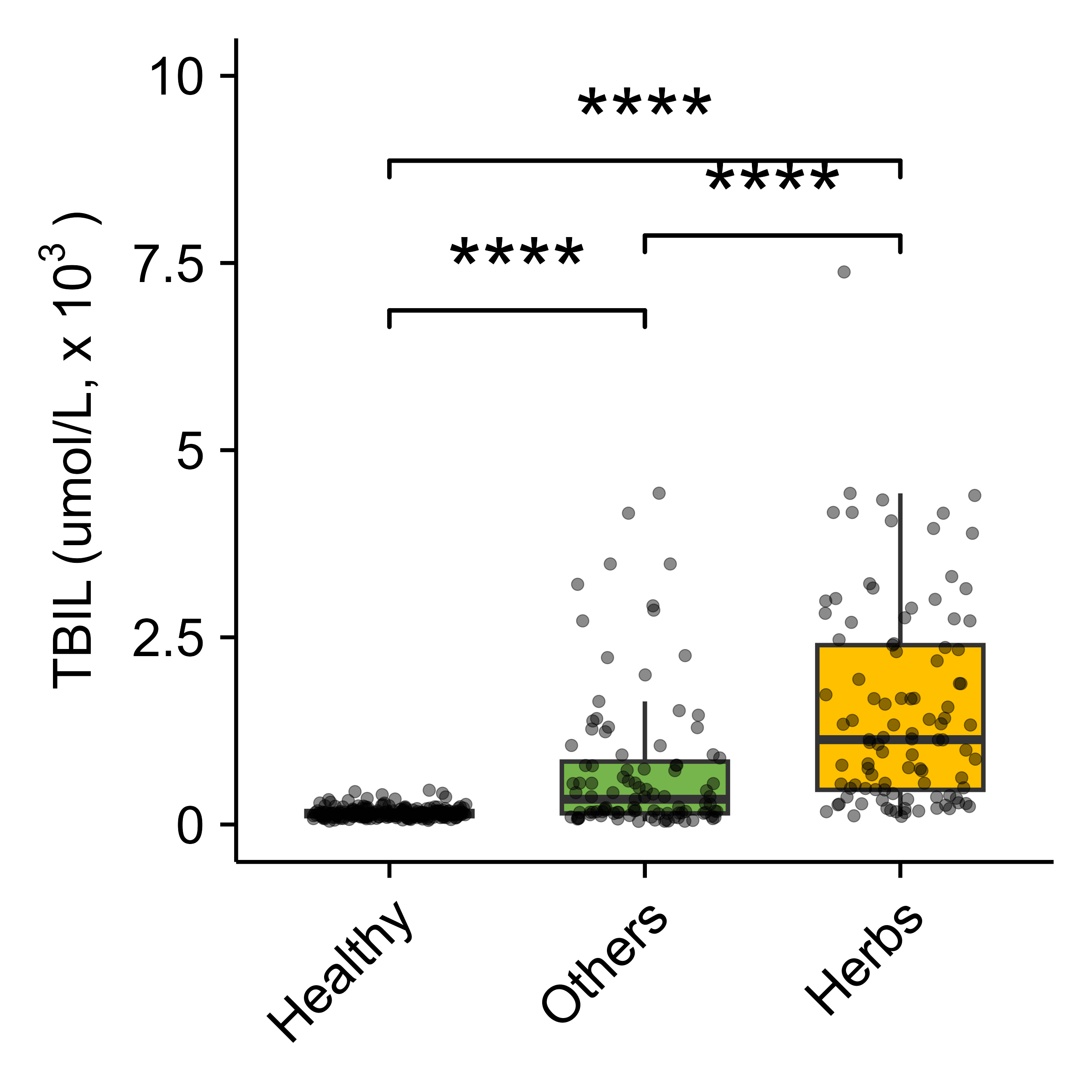


**Fig. S2.** The levels of total bilirubin (TBIL) in the serum samples of healthy controls, herbs-DILI, and others-DILI patients. *, *P* < 0.05; **, *P* < 0.01; ***, *P* < 0.001; ****, *P* < 0.0001; ns, not significant.


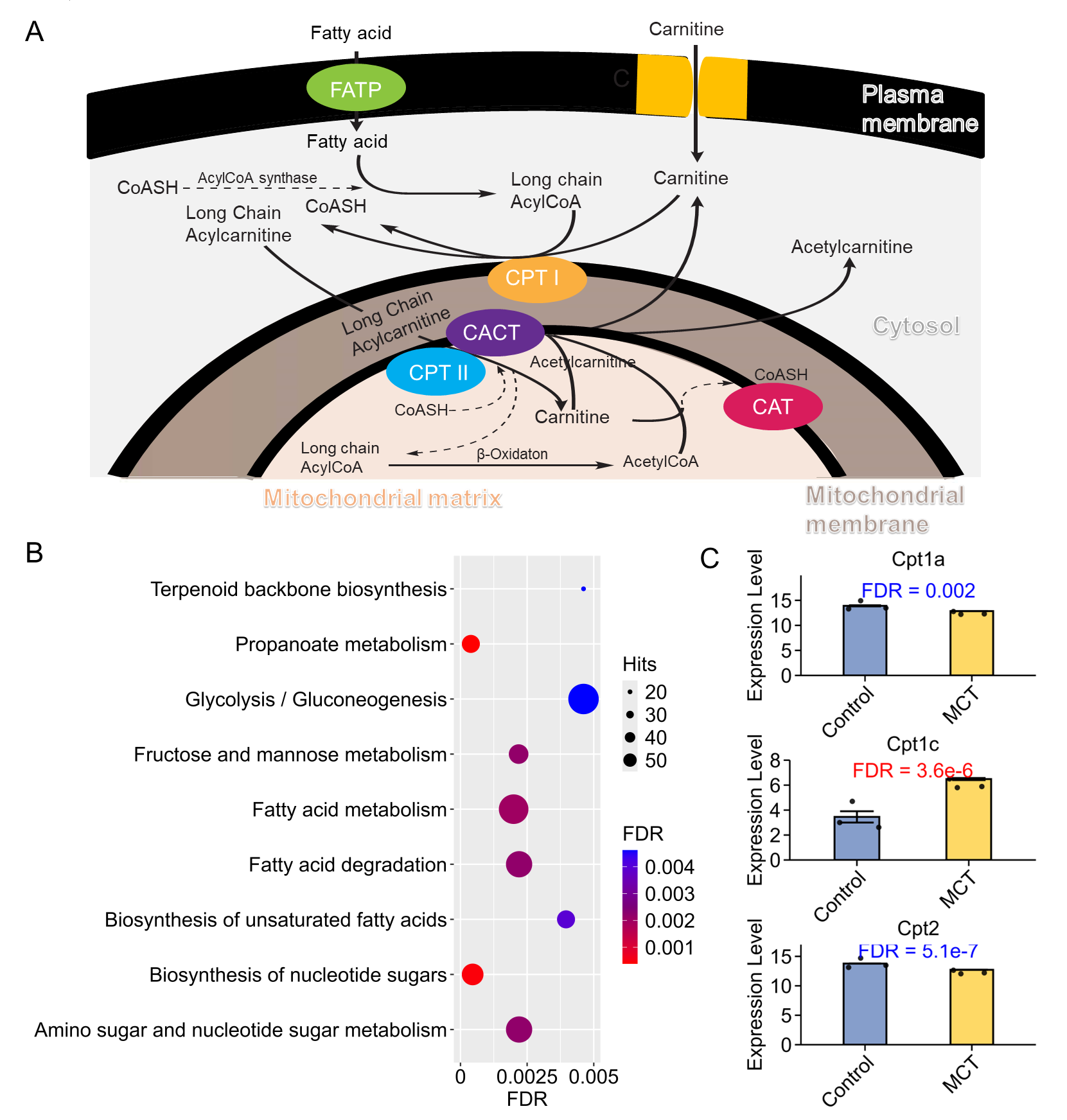


**Fig. S3.** Carnitine metabolism in GSE102150 transcriptome dataset.

(A) Outlet for carnitine metabolism pathway. (B) The significant pathways (adjusted *P* < 0.005) enriched with the differentially expressed genes in the liver tissues of monocrotaline-induced liver injury mouse models in GEO102150 dataset. (C) The mRNA expressions of carnitine acyltransferase (Cpt) 1a, Cpt1c, and Cpt2 in the liver tissues of monocrotaline (MCT)-induced liver injury mouse models in GEO102150 dataset.


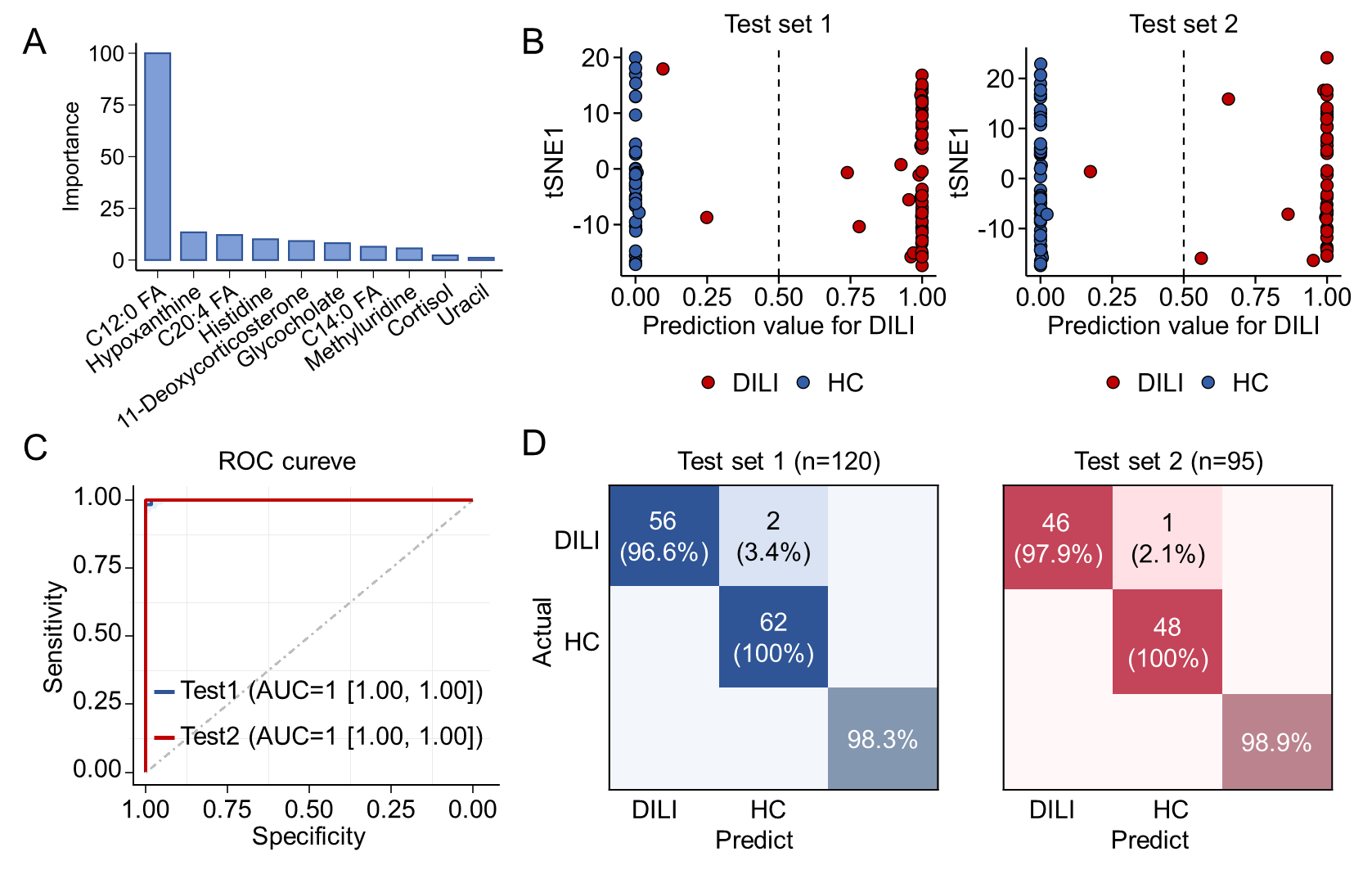


**Fig. S4.** Machine learning-derived prediction model based on serum metabolome for drug-induced liver injury (DILI) diagnosis.

(A) Contribution of the ten metabolites to the 10-differential metabolites (DM) model. The ten metabolites are C12:0 FA, hypoxanthine, C20:4 FA, histidine, 11-deoxycorticosterone, glycocholate, C14:0 FA, methyluridine, and uracil. (B) The dotted line representing the cutoff value of 0.50 used to separate the predicted healthy controls (HC) from DILI on test sets 1 and 2. (C) The Receiver operating characteristic (ROC) curve and area under the curve (AUC) values for identifying DILI patients from different drug-sources on the test set 1 and 2. A 95% confidence interval was computed with 2000 stratified bootstrap replicates. (D) Confusion Matrix of the 10-DM model for discriminating HC and DILI samples on the test set 1 and 2.


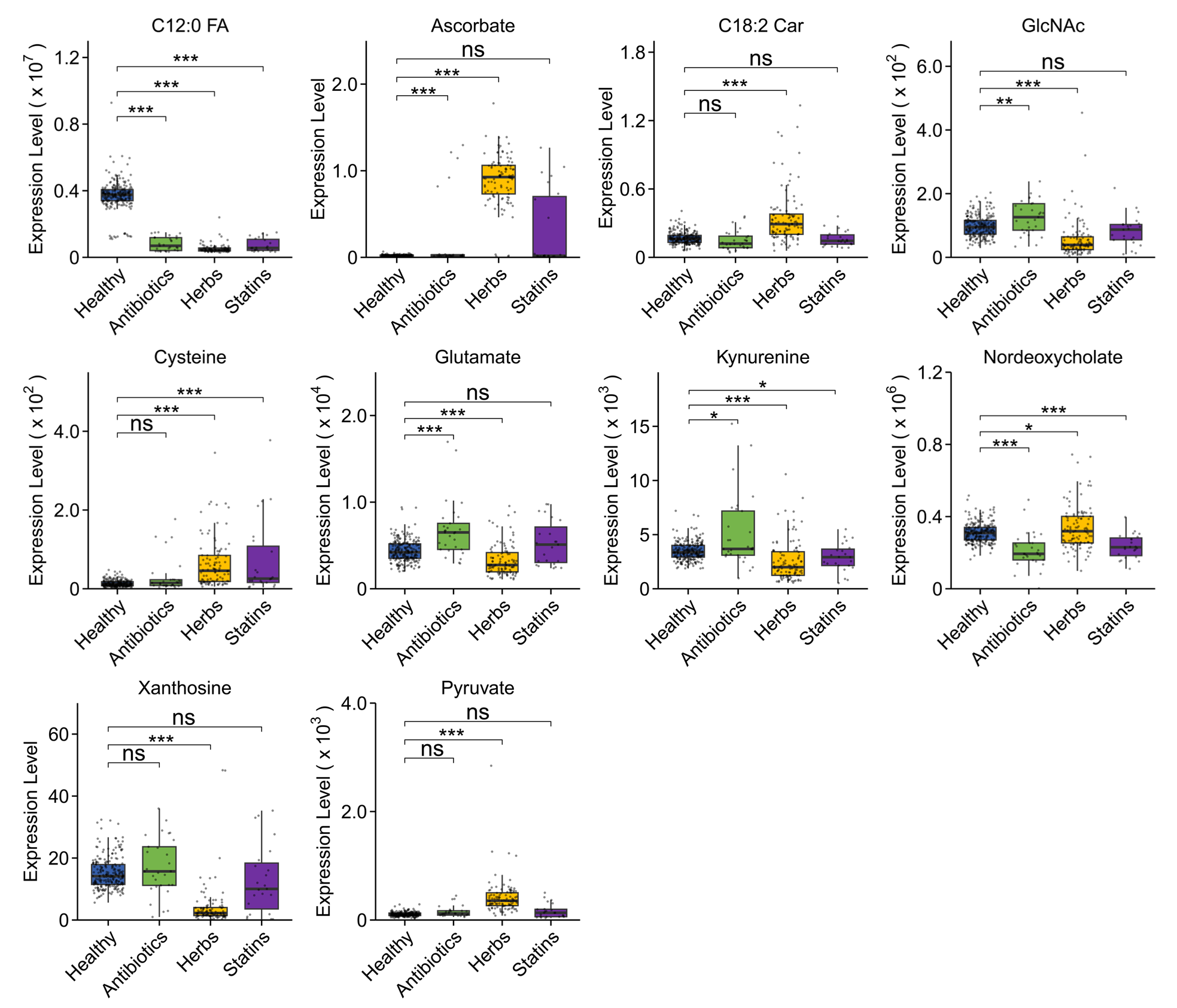


**Fig. S5.** The expressions of the ten metabolites selected for model development in the serum samples of healthy controls, antibiotics-DILI, herbs-DILI, and statins-DILI patients. The ten metabolites are C12:0 FA, ascorbate, C18:2 Car, pyruvate, kynurenine, nordeoxycholate, cysteine, xanthosine, glutamate, and N-Acetyl-D-glucosamine (GlcNAc). mean ± SEM. *, *P* < 0.05; **, *P* < 0.01; ***, *P* < 0.001; ns, not significant.
